# The Selective WEE1 Inhibitor Azenosertib Shows Synergistic Antitumor Activity with *KRAS*^G12C^ Inhibitors in Preclinical Models

**DOI:** 10.1158/2767-9764.CRC-24-0411

**Published:** 2025-02-05

**Authors:** Nathan M. Jameson, Daehwan Kim, Catherine Lee, Blake Skrable, Alexandra Shea, Xiao Guo, Hooman Izadi, Mona Abed, Olivier Harismendy, Jianhui Ma, Doris S. Kim, Mark R. Lackner

**Affiliations:** Zentalis Pharmaceuticals, Inc., San Diego, California.

## Abstract

Kirsten rat sarcoma (KRAS) is a potent oncogenic driver that results in the downstream hyperactivation of MAPK signaling while simultaneously increasing replication stress (RS) and accumulation of DNA damage. *KRAS*^G12C^ mutations are common and targetable alterations. Therapeutic inhibition of *KRAS*^G12C^ and eventual resistance to these inhibitors are also known to drive RS and DNA damage through adaptive mechanisms that maintain addiction to high MAPK signaling. High levels of RS result in a stronger reliance on cell-cycle checkpoints, thereby introducing a vulnerability to inhibition of cell-cycle checkpoint regulators such as the WEE1 kinase. This provides a rationale for combining azenosertib, a novel, selective, and orally bioavailable WEE1 inhibitor, with *KRAS*^G12C^ inhibitors. An *in vitro* combination of azenosertib with multiple *KRAS*^G12C^ inhibitors demonstrated synergistic cell growth inhibition across a panel of *KRAS*^G12C^ cell lines in both 2D and 3D assays. *In vivo* studies demonstrated that azenosertib exhibited significant monotherapy activity as well as synergistic tumor growth inhibition (TGI) when combined with *KRAS*^G12C^ inhibitors, including tumor regression in cell-derived xenograft models of non–small cell lung cancer, colorectal cancer, and pancreatic ductal adenocarcinoma. Importantly, *KRAS*^G12C^ inhibitor–resistant cell-derived xenograft and patient-derived xenograft models demonstrated synergistic TGI in combination arms. Finally, analysis of biomarkers from *in vitro* and *in vivo* tumor samples displayed increases in protein markers of RS, DNA damage, and apoptosis after combination treatment. Taken together, our results suggest that the combination of azenosertib with *KRAS*^G12C^ inhibitors enhances TGI over single-agent therapy and may be an effective treatment strategy for patients with *KRAS*^G12C^ tumors.

**Significance:**

Resistance to *KRAS*^G12C^ inhibitors is a growing clinical concern. The synergistic interaction observed between azenosertib and multiple *KRAS*^G12C^ inhibitors could result in deeper and more durable responses.

## Introduction

Kirsten rat sarcoma (KRAS) is a member of the RAS family of proteins that function as key cellular switches, integrating upstream signals from growth factor receptors and transmitting these signals downstream to drive cell growth and proliferation. *RAS* mutations, most commonly in codons 12 and 13 of exon 2, act to preferentially maintain the GTP-bound active “on” state of KRAS, driving downstream growth signaling independent of upstream activation (1). *KRAS*^G12C^ alterations are the most common *KRAS* mutation in non–small cell lung cancer (NSCLC), representing ∼10% to 13% of patients with advanced NSCLC (2). *KRAS*^G12C^ is also present in other tumor types including ∼3% of colorectal cancer, ∼1% of pancreatic ductal adenocarcinoma (PDAC), and ∼1% to 3% of other solid tumors (3). Historically, the presence of a *KRAS*^G12C^ mutation was a negative prognostic factor, highlighting the lack of effective treatment options for these patients (4, 5). Long thought to be undruggable, the discovery of a novel allosteric pocket near the mutated cysteine of *KRAS*^G12C^ enabled the development and clinical validation of novel targeted therapies (6), which ultimately resulted in the FDA approval of both sotorasib and adagrasib in patients with previously treated *KRAS*^G12C^ NSCLC (7, 8). Although responses to these agents are robust, some patients are inherently refractory and almost all responders eventually acquire resistance (9, 10). Resistance mechanisms are variable; however, most result in the reactivation of MAPK signaling through mutation, amplification, or other nongenetic mechanisms (11, 12). To address these resistance mechanisms, a wide variety of combination strategies have been implemented both preclinically and clinically to eliminate escape pathways, many of which target components upstream (EGFR, SHP2, and SOS1) or downstream (RAF, MEK, and ERK) of RAS (13). Less frequent but equally rational are strategies which capitalize on other inherent vulnerabilities such as replication stress (RS), chromosomal instability (CIN), and loss of cell-cycle checkpoint control (14).

It has been well established that oncogenic drivers, including KRAS, increase DNA damage and RS through modulation of a wide variety of cellular processes including DNA replication, DNA damage response, and cell-cycle checkpoint control (15–20). Cancer cells with these driver mutations have adapted to tolerate higher levels of RS; however, some studies suggest that further exacerbation of RS and DNA damage may push these cells past manageable levels of RS and DNA damage and, ultimately, result in mitotic catastrophe and apoptosis (21, 22). Inhibition of these oncogenic drivers might be hypothesized to reduce these RS phenotypes, yet paradoxically it has also been demonstrated that inhibition of oncogenic drivers like KRAS can drive high levels of CIN and RS (17, 23, 24). Through a phenomenon known as oncogene addiction, cancer cells can become dependent upon activation of a single oncogenic pathway, and although blocking signaling of this pathway can inhibit tumor cell growth, it can also prompt cells to increase CIN and RS as an adaptive mechanism (25, 26).

High levels of RS result in a stronger reliance on cell-cycle checkpoints, thereby introducing a vulnerability to inhibition of cell-cycle checkpoint regulators such as WEE1. WEE1 is a tyrosine kinase that plays a role in several stages of the cell cycle, including proper regulation of the G_1_–S cell-cycle transition and protection of stalled replication forks through inhibition of CDK2 and the G_2_–M transition through inhibition of CDK1 (27). Tight regulation of cell-cycle checkpoints by WEE1 allows for nonmalignant cells to pause the cell cycle and repair damaged DNA. Cell-cycle checkpoint defects including *TP53* mutation are common across cancer types and allow for cell-cycle progression in the presence of DNA damage, CIN, and RS, enabling adaptation to treatment and persistence in response to targeted therapies and chemotherapy (16, 17, 28, 29). Inhibition of WEE1 drives these cancer cells to enter unscheduled mitosis without adequate DNA repair, eventually triggering cell death as these levels of DNA damage become intolerable (30). Due to the important role of WEE1 in G_1_–S and G_2_–M cell-cycle checkpoints, high levels of baseline RS and DNA damage in *KRAS*-mutant cancer cells may confer sensitivity to WEE1 inhibition. Indeed, WEE1 has been shown to be a key dependency in *KRAS*-mutant cancers (20, 21, 31–34), and co-inhibition of KRAS with WEE1 and other cell-cycle and mitotic proteins has been identified as promising combination strategies through large-scale screening efforts (32, 35, 36).

Given the high levels of RS and DNA damage in treatment-naïve and on-treatment *KRAS*-mutant cancer cells, we hypothesize that the combination of *KRAS*^G12C^ inhibition with azenosertib, a novel, selective, and orally bioavailable inhibitor of WEE1 (37), could capitalize on these inherent vulnerabilities and result in combination benefit in *KRAS*^G12C^ cancer models. In this study, we examine combination treatment of azenosertib with sotorasib or adagrasib and their effects on cancer cell growth inhibition *in vitro* and *in vivo*. We show that combination treatment drives synergistic tumor growth inhibition (TGI) in 2D and 3D *in vitro* assays across multiple indications. In *KRAS*^G12C^ inhibitor–sensitive cell-derived xenograft (CDX) and patient-derived xenograft (PDX) models of NSCLC, colorectal cancer, and PDAC, we show that combination treatment results in deeper and more durable tumor responses than either single agent alone. We also demonstrate synergy in *KRAS*^G12C^ inhibitor–resistant CDX and PDX models. Finally, we observe increases in pharmacodynamic (PD) biomarkers of DNA damage and apoptosis with the combination over monotherapy.

## Materials and Methods

### Cell culture and reagents

RPMI-1640 (#30-2001) was obtained from the ATCC. DMEM (#12430-054), GlutaMAX supplement (#35050-061), penicillin and streptomycin (P/S; #15140-122), and heat-inactivated FBS (#A38400-02) were obtained from Gibco/Thermo Fisher Scientific. NCI-H358 (RRID: CVCL_1559), NCI-H23 (RRID: CVCL_1547), NCI-H1792 (RRID: CVCL_1495), NCI-H2030 (RRID: CVCL_1517), and NCI-H2122 (RRID: CVCL_1531) were maintained in RPMI +10% FBS +1% P/S. SW1573 (RRID: CVCL_1720) was maintained in RPMI +1% GlutaMAX + 10% FBS + 1% P/S. SW837 (RRID: CVCL_1729), SW1463 (RRID: CVCL_1718), and MIA PaCa-2 (RRID: CVCL_0428) were maintained in DMEM +10% FBS +1% P/S. All cell lines were obtained from the ATCC. Human cancer cell lines were maintained at 37°C in a humidified incubator at 5% CO_2_ in complete culture media as recommended either by ATCC or in published studies. Cell lines were carried for no more than 20 cell passages. Cell line integrity was confirmed by short tandem repeat and *Mycoplasma* testing at IDEXX.

### Formulations

Azenosertib was synthesized at Pharmaron. Sotorasib, adagrasib, and RMC-6236 were purchased from Synnovator, Inc. (#SYNB20447, #SYNB22764, and #RMC-6236) for *in vitro* studies or from MedChemExpress (#HY-114277 and #HY-130149) for *in vivo* studies. For *in vitro* studies, compounds were resuspended in DMSO as 10 mmol/L stocks. For use *in vivo*, azenosertib was prepared in 20% 2-hydroxypropyl-β-cyclodextrin in water, sotorasib was prepared in 2% hydroxypropyl methylcellulose + 1% Tween-80 in water, and adagrasib was prepared in 10% Captisol in 50 mmol/L citrate buffer pH = 5.0. Vehicle controls in all studies were 20% 2-hydroxypropyl-β-cyclodextrin in water.

### *In vitro* assays

For determining 2D synergy, 1,500 cells in 100 μL complete culture media were deposited in the center wells of 96-well white-walled plates (Corning Life Sciences, #3903). For determining 3D synergy, 1,500 cells in 100 μL complete culture media were deposited into the center wells of an ultralow attachment (ULA) U-bottom 96-well plate (S-BIO, #MS9096UZ). The plates were incubated for 24 hours to allow for spontaneous spheroid formation. Successfully formed spherical structures with regular edges that proliferated over time (as determined visually under a microscope at 10× magnification under bright-field imaging) were chosen to proceed for downstream analysis. Twenty-four hours after seeding, compounds were deposited using an automated drug dispenser (Pico 8, Thermo Fisher Scientific) at the indicated concentrations. Total DMSO content was normalized to 0.1% total volume in all wells. Cell viability was assessed after 7 days using CellTiter-Glo (Promega, 2D: #G7573, 3D: #G9682) according to the manufacturer’s instructions. For 3D spheroid growth assays, spheroids were imaged with the Incucyte SX5 instrument (Sartorius) every 8 hours. Synergy was determined with the SynergyFinder tool (http://www.synergyfinderplus.org/; ref. 38) using the Loewe synergy model. Loewe values >10 indicate synergistic combination effects of the drugs, Loewe values from −10 to 10 indicate additive effects, and Loewe values < −10 indicate antagonism.

#### siRNA transfection

Cells were deposited in a 96-well plate incubated overnight. Transfection was performed with siControl (Silencer Select Negative Control No. 2, # 4390847) or siWEE1 (Dharmacon, #L-005050-00-0005) the following day using Lipofectamine RNAiMAX (Thermo Fisher Scientific, #13778150) following the manufacturer’s protocol. Immediately after transfection, compounds were dispensed into the 96-well plate using Pico 8. After 72 hours, cell viability was assessed using the 2D CellTiter-Glo reagent. Transfected cells were collected and lysed on ice in RIPA lysis buffer (Thermo Fisher Scientific, #89901), and protein concentrations were determined using the Pierce BCA Protein Assay kit (Thermo Fisher Scientific, #23227). Western blotting was performed using the Jess Simple Western system (ProteinSimple) according to the manufacturer’s standard method for the 12 to 230 kDa Jess Separation Module (#SM-W004).

#### Cell-cycle analysis

Cells were seeded in a 12-well plate, allowed to attach overnight, and then treated with the indicated inhibitors for 24 hours. The cells were then pulsed with 10 μmol/L EdU (Click-iT Plus EdU Alexa Fluor 647, Thermo Fisher Scientific, #C10634) at 37°C for 2 hours, harvested, washed with 1% BSA in PBS, fixed with Click-iT fixative, and permeabilized with 1× Click-iT permeabilization and wash reagent. Click-iT EdU was detected by incubating the cells in Click-iT Plus reaction cocktail at room temperature for 30 minutes before DNA content staining (FxCycle Violet Stain, Thermo Fisher Scientific, #F10347). Samples were analyzed using an Attune flow cytometer, and data analysis was performed using FlowJo and GraphPad Prism.

### Biomarker analysis

For 2D biomarker analysis, cells were lysed 24 hours after treatment with RIPA buffer (Thermo Fisher Scientific, #89900) and sonicated on a Bioruptor Pico instrument (Diagenode). For 3D biomarker analysis, cells were incubated for 120 hours in ULA plates to allow for spheroid formation and then treated for 24 hours. A total of 96 spheroids per treatment condition were pooled, lysed in RIPA buffer, and sonicated. For biomarker analysis of mouse tissue samples, snap-frozen tumor tissues were lysed in RIPA buffer using a tissue homogenizer (BeadBlaster 24R, Benchmark Scientific). All lysis buffers were supplemented with protease and phosphatase inhibitors and quantified by bicinchoninic acid assay.

Western blotting was performed using Jess. Chemiluminescence reactions with antibodies were measured, and their digital blot images were constructed using Compass software (version 6.2). Quantification by densitometry was performed using the area of targeted proteins and normalized to the total protein. The list of the antibodies used is reported in Supplementary Table S1.

### *In vivo* xenograft studies

CDX/PDX mouse studies were conducted at the following contract research organizations: Pharmaron Inc., Crown Bioscience, and XenoSTART. Procedures related to animal handling, care, and treatment complied with all applicable regulations and guidelines of the institutional animal care and use committee at each facility. Studies used 6- to 12-week-old female immunodeficient mice purchased from Shanghai Model Organisms, GemPharmatech Biotech Co., Beijing Anikeeper Biotech Co., or Charles River Laboratories.

For CDX models, mice were inoculated on the right flank with tumor cells (5 × 10^6^–2 × 10^7^) in 100 to 200 μL of media or media supplemented with Matrigel (1:1). For the NCI-H2030-R CDX model, cryopreserved tumor fragments were thawed and implanted subcutaneously for initial tumor development and subsequently passaged in mice maintained on 100 mg/kg sotorasib for tumor expansion. When tumors reached ∼2,000 mm^3^, they were passaged again into a final set of study mice and treated with 100 mg/kg sotorasib until grouping.

PDX models were generated from patients in accordance with protocols approved by the hospital’s institutional ethical committee. XenoSTART PDX models were established from the samples collected from informed-consented patients being cared for at the START Center for Cancer Care and the Methodist Health System, both located in San Antonio, Texas. Recovered tumor fragments were implanted into immunodeficient mice, and treatment started when the average tumor volume reached 150 to 300 mm^3^. Tumor fragments from stock mice were harvested and used for inoculation into mice.

Animals were randomized into groups (*n* = 3–10/group). Compounds were administered daily via oral gavage at 10 mL/kg. Mouse bodyweights and tumors were measured twice weekly. Tumors were measured using digital calipers, and tumor volume in mm^3^ was calculated using the formula volume = [(width)^2^ × length]/2. Percent change in tumor volume (ΔTV) for individual mice was calculated and plotted using the formula [(TV_d_ − TV_0_)/TV_0_] × 100. Percentage change in body weight for each animal on a given day was determined as BW change (%) = [(BW_d_ − BW_0_)/BW_0_] × 100. TGI was calculated using the formula [1 − (T_d_ − T_0_)/(C_d_ − C_0_)] × 100%. Percent treated/control was calculated using the formula [(T_d_ − T_0_)/(C_d_ − C_0_)] × 100%. Animals were terminated early if tumor burden reached 2,000 mm^3^ or if adverse effects were observed, with body weight loss >20% as a surrogate.

For the mouse PD study, tumors were harvested 24 hours after single dose. Tumor tissue was snap-frozen in liquid nitrogen or formalin-fixed and embedded in paraffin for further analysis. Ki67 IHC was performed on 5-μm sections. Antigen retrieval was performed with citrate buffer pH 6.0 and blocked with goat serum. Primary rabbit polyclonal Ki67 was stained for 1 hour (Supplementary Table S1) and detected with a goat anti–rabbit horseradish peroxidase secondary antibody. IHC H-scoring and hematoxylin and eosin interpretation were performed by a board-certified veterinary pathologist with experience in laboratory animals and toxicologic pathology. Images had three areas counted for the percentage of positive cells in each of the three 40× magnification fields.

### Statistical analysis

Unless stated otherwise, all data were statistically analyzed using GraphPad Prism software (version 10). Grouped data were analyzed by two-way repeated measures (RM) ANOVA. Data are presented as mean ± SEM. Results were considered statistically significant if *, *P* ≤ 0.05; **, *P* ≤ 0.01; ***, *P* ≤ 0.001.

### Data availability

All data generated or analyzed during this study are included in this article or will be made available from the corresponding author upon reasonable request.

## Results

### Combination of azenosertib and *KRAS*^G12C^ inhibitors demonstrates synergy in 2D cellular assays

**Figure 1:**
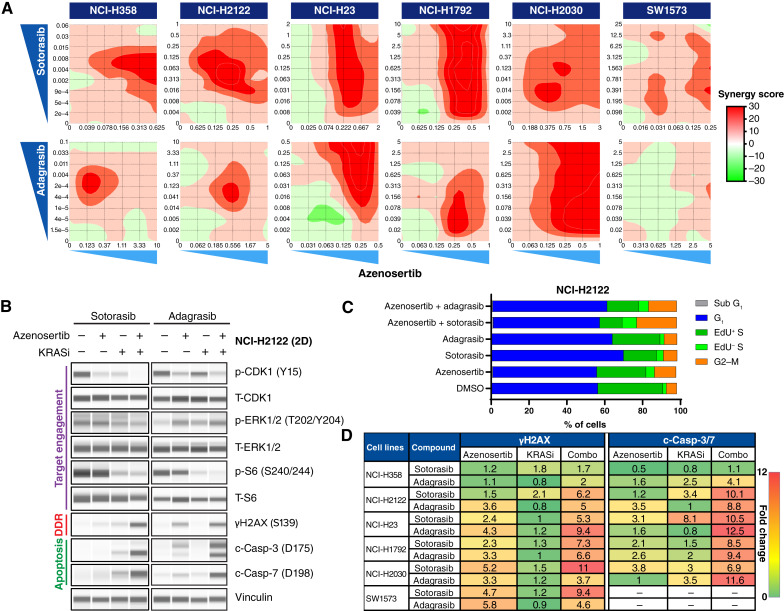
Combination of azenosertib and *KRAS*^G12C^ inhibitors demonstrates synergy in NSCLC in 2D *in vitro* cellular assays. **A,** Seven-day combination treatment dose matrices in *KRAS*^G12C^ NSCLC cell lines cultured in 2D. The Loewe additivity model is depicted in which scores ≥10 are synergistic. Doses are expressed in μmol/L. Cell lines are arranged from most sensitive to least sensitive to *KRAS*^G12C^ monotherapy (left to right). **B,** Western blots of protein expression from NCI-H2122 NSCLC cells treated with DMSO, 250 nmol/L azenosertib, 62.5 nmol/L sotorasib, 25 nmol/L adagrasib, or the combination for 24 hours. **C,** DNA content analysis by flow cytometry and represented as the percentage of total cells in each cell-cycle phase. EdU^+^ and EdU^−^ DNAs distinguish active vs. stalled DNA replication. NCI-H2122 NSCLC cells were treated with DMSO, 500 nmol/L azenosertib, 62.5 nmol/L sotorasib, 50 nmol/L adagrasib, or the combination for 24 hours. **D,** Quantification of γH2AX and c-Casp-3/7 protein levels from cell lines treated for 24 hours based on doses associated with the highest calculated Loewe synergy score. SW1573 did not have quantifiable intensities for c-Casp-3/7. Cell lines are ordered by *in vitro* sensitivity to *KRAS*^G12C^ inhibition (most sensitive top). Results are expressed as fold change relative to DMSO control. DDR, DNA damage response. KRASi, KRAS inhibitor.

To begin investigating the hypothesized synergy between inhibition of WEE1 and *KRAS*^G12C^, we assembled a panel of NSCLC cell lines with varying levels of sensitivity to both azenosertib and the *KRAS*^G12C^ inhibitors sotorasib and adagrasib. We tested the sensitivity of each cell line to each compound individually and then chose a range of doses to test in combination in a matrixed format. We found that all cell lines tested exhibited synergy, defined by Loewe synergy scores >10, regardless of their sensitivity to either WEE1 or *KRAS*^G12C^ inhibitor monotherapy, and the synergy was similar between the two *KRAS*^G12C^ inhibitors tested (Fig. 1A). Although Loewe synergy scores are commonly utilized to indicate synergy, the Bliss independence model can also be utilized for combination studies in which each compound acts on independent signaling pathways (39). Bliss synergy scores for each combination demonstrated similar patterns of synergy to Loewe scores, indicating that this synergy phenotype is independent of statistical methodology (Supplementary Fig. S1).

To understand the mechanism of this observed synergy, we analyzed PD biomarker changes in response to azenosertib and *KRAS*^G12C^ inhibitor monotherapy and combination treatment in the NCI-H2122 NSCLC cell line (Fig. 1B). WEE1 phosphorylates CDK1 at tyrosine 15 (p-CDK1 Y15), and the loss of this phosphorylation signal is a measure of target engagement by azenosertib. We found that p-CDK1 Y15 was reduced in response to azenosertib, demonstrating suppression of WEE1 kinase activity. Surprisingly, treatment with either sotorasib or adagrasib alone also reduced p-CDK1 Y15, possibly due to an extended G_1_-phase as reported previously (40). Moreover, combination treatment further reduced p-CDK1 Y15 levels, demonstrating additional loss of G_2_–M and G_1_–S cell-cycle checkpoint control. We found that the downstream KRAS pathway targets p-ERK1/2 T202/Y204 and p-S6 S240/244 were both reduced in response to treatment with *KRAS*^G12C^ inhibitor monotherapy, as well as the combination with azenosertib, demonstrating on-target inhibition of KRAS signaling. Furthermore, we observed a modest increase in levels of H2AX at S139 (γH2AX), a DNA damage marker and proxy for RS, in response to either monotherapy, indicating that both inhibitors can individually upregulate some level of DNA damage and RS. Critically, combination treatment resulted in a much stronger upregulation of γH2AX and apoptosis as shown by cleaved caspase 3 (c-Casp-3) and c-Casp-7 (Fig. 1B). Cell-cycle analysis by DNA content revealed increased G_2_–M phase arrest in response to azenosertib monotherapy treatment, and this effect was exacerbated in the combination. Increased DNA replication fork stalling was also observed (as marked by EdU-DNA staining) with monotherapy azenosertib or combination treatment, consistent with the role of WEE1 in replication fork stability (Fig. 1C; ref. 27).

To determine if PD changes were cell line–specific, we expanded the analysis of γH2AX and c-Casp-3/7 to the entire panel of NSCLC cell lines. Consistent with what was observed in NCI-H2122 cells, we detected mild to moderate upregulation of γH2AX and c-Casp-3/7 in response to either monotherapy treatment in most cell lines. Importantly, combination treatment resulted in markedly higher γH2AX and c-Casp-3/7 signals across the majority of models tested, confirming the synergistic effect of azenosertib with *KRAS*^G12C^ inhibitors to drive DNA damage, RS, and apoptosis (Fig. 1D). The magnitude of increases in γH2AX and c-Casp-3/7 varied across different cell lines, underscoring the heterogeneity of response to both monotherapy and combination treatment; however, in 22 of 24 (92%) conditions measured, the magnitude of PD changes in response to combination treatment was greater than monotherapy.

Although *KRAS*^G12C^ is most frequently observed in NSCLC, it is also detected in other tumor types including PDAC and colorectal cancer. We therefore sought to evaluate whether the synergistic effect of azenosertib and *KRAS*^G12C^ inhibitors applies to indications beyond NSCLC. Consistent with the observations in NSCLC, matrixed combination treatment with azenosertib and *KRAS*^G12C^ inhibitors resulted in synergy in all tested colorectal cancer and PDAC cell lines (Supplementary Fig. S2A). Similarly, downstream biomarker changes in target engagement, DNA damage, and apoptosis were consistent with those observed in NSCLC cell lines (Supplementary Fig. S2B), indicating that the mechanism was applicable across NSCLC, colorectal cancer, and PDAC. We further found that the synergistic cell growth inhibition is due to the specific targeting of WEE1 by azenosertib as the knockdown of WEE1 by siRNA combined with *KRAS*^G12C^ inhibition resulted in increased synergy when compared against siControl (Supplementary Fig. S2C). Finally, we combined azenosertib with RMC-6236, a pan-RAS (on) inhibitor with activity in *KRAS*^G12C^ and other activating *RAS* mutations (41) and found strong synergy in the NCI-H1792 cell line model (Supplementary Fig. S2D).

### Combination of azenosertib with *KRAS*^G12C^ inhibitors reduces tumor cell growth and induces DNA damage and apoptosis in 3D cellular assays

**Figure 2:**
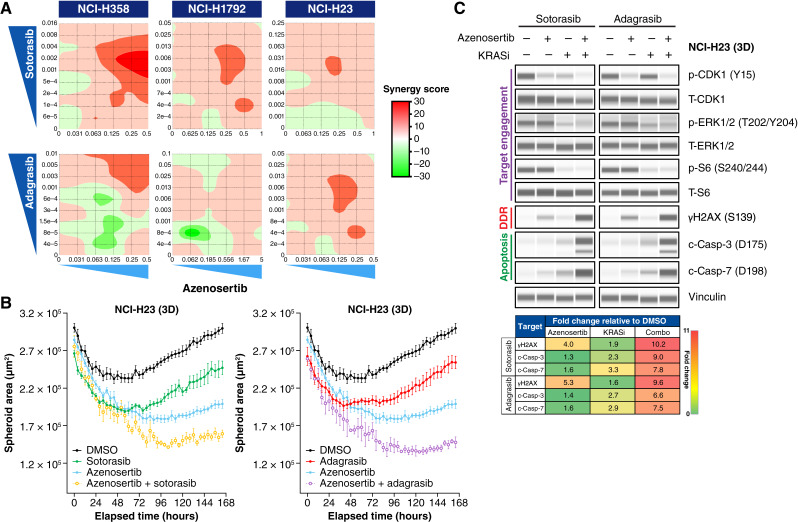
Combination of azenosertib with *KRAS*^G12C^ inhibitors reduces tumor cell growth and induces DNA damage and apoptosis *in vitro* in 3D. **A,** Seven-day combination treatment dose matrices in *KRAS*^G12C^ NSCLC cell lines cultured as 3D spheroids. Cell viability was measured by 3D CellTiter-Glo and analyzed using the SynergyFinder tool. The Loewe additivity model is depicted in which scores ≥10 are synergistic. Doses are expressed in μmol/L. Cell lines are arranged from most sensitive to least sensitive to *KRAS*^G12C^ monotherapy (left to right). **B,** Longitudinal cell growth analysis of NCI-H23 spheroids treated with DMSO, 125 nmol/L azenosertib, 5 nmol/L sotorasib, or 5 nmol/L adagrasib for 7 days. The spheroid area was imaged and calculated every 8 hours using an Incucyte instrument. *P* < 0.0001 for all comparisons with vehicle; *P* < 0.0001 for combination compared with monotherapies. **C,** Top, Western blot of protein expression from NCI-H23 spheroids treated with DMSO, 260 nmol/L azenosertib, 38 nmol/L sotorasib, or 20 nmol/L adagrasib for 24 hours. Bottom, Quantification of γH2AX and c-Casp-3/7 protein levels in response to treatment. Results are expressed as fold change relative to DMSO control. DDR, DNA damage response. KRASi, KRAS inhibitor.

Although the 2D cell-based studies described above suggest a synergistic relationship between inhibition of WEE1 and *KRAS*^G12C^ in *KRAS*^G12C^-driven tumors, previous studies have demonstrated that RAS-driven models grown as 3D spheroids may more accurately reflect the sensitivity to *KRAS*^G12C^ inhibition *in vivo* (42)*.* We therefore sought to validate the 2D synergy findings in a subset of cell lines that could be cultured as anchorage-independent spheroids under ULA conditions. We found that combination treatment of NCI-H358, NCI-H1792, and NCI-H23 spheroids displayed synergy under 3D conditions, confirming the synergistic effect of that was detected in 2D cultures (Fig. 2A).

To directly visualize how these 3D spheroid models responded to treatment, we performed live cell imaging–based spheroid growth assays (Fig. 2B). When compared with the DMSO control, either monotherapy resulted in statistically significant (*P* < 0.0001) reduction in spheroid size. Additionally, combination treatment resulted in an even greater reduction of spheroid size when compared against either monotherapy (*P* < 0.0001). These data provide a functional demonstration of tumor cell growth inhibition under anchorage-independent conditions in response to combination treatment.

Next, we interrogated biomarker changes in the spheroid growth environment in response to treatment. Similar to 2D conditions, we found that NCI-H23 spheroids treated with azenosertib monotherapy resulted in downregulation of p-CDK1 Y15 and upregulation of γH2AX and c-Casp-3/7. Similarly, we found that monotherapy treatment with either sotorasib or adagrasib resulted in downregulation of p-CDK1 Y15, p-ERK1/2, and p-S6, and combination treatment resulted in marked increases in γH2AX and c-Casp-3/7 and further downregulation of p-CDK1 Y15 relative to monotherapy (Fig. 2C).

These data further demonstrate the *in vitro* synergistic activity of WEE1 and *KRAS*^G12C^ inhibition through increased DNA damage, RS, and apoptosis in orthogonal and more physiologically relevant model systems.

### Treatment of an NSCLC CDX model with azenosertib + sotorasib results in tumor regressions and increased DNA damage and apoptosis *in vivo*

**Figure 3:**
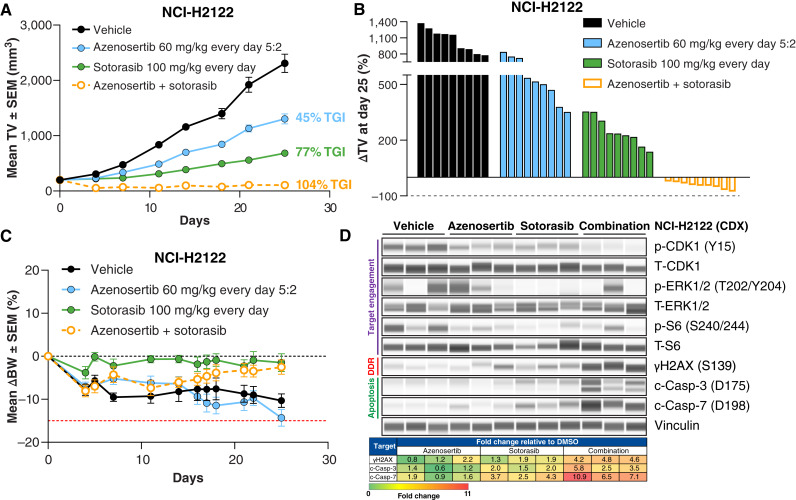
Treatment of an NSCLC CDX model with azenosertib + sotorasib results in tumor regressions and increased DNA damage and apoptosis *in vivo*. **A,** Mean tumor volume ± SEM of subcutaneous NCI-H2122 xenografts in NOD/SCID mice treated for 25 days (*n* = 9/group). *P* < 0.0001 for all comparisons with vehicle; *P* < 0.0001 for combination compared with monotherapies. **B,** Percent change in tumor volume (ΔTV) of individual mice on day 25 of treatment. Values below 0 indicate regression. **C,** Mean percent change in body weight relative to day 0 (ΔBW) ± SEM. NCI-H2122 was noted to be cachexic in NOD/SCID mice (seen by minor weight loss in the vehicle group), but treatments were well tolerated and did not exacerbate weight loss relative to vehicle. Red dashed line indicates −15% cutoff in weight change. **D,** Top, Western blots of protein expression from NCI-H2122 tumors treated with one dose of the indicated compounds. Bottom, Quantification of γH2AX and c-Casp-3/7 protein levels in response to treatment. Results are expressed as fold change relative to DMSO control. DDR, DNA damage response.

Although the combination of WEE1 and *KRAS*^G12C^ inhibitors showed activity *in vitro*, interrogation in animal models may provide additional translational evidence of synergy. We treated mice bearing NCI-H2122 CDX with clinically relevant dosing regimens. Monotherapy treatment with azenosertib once daily for 5 days on and 2 days off (every day 5:2) or sotorasib once daily resulted in moderate but statistically significant TGI (45% and 77%, respectively; *P* < 0.0001 vs. vehicle; Fig. 3A). Combination treatment resulted in 104% TGI and led to tumor regressions in all nine animals (*P* < 0.0001 vs. either monotherapy; Fig. 3A and B). Of note, azenosertib and sotorasib were well tolerated over the course of the study as illustrated by minimal changes in mean body weight relative to that of the vehicle group (Fig. 3C).

To assess PD modulation *in vivo*, we performed a separate PD study in NCI-H2122 and analyzed tumors from animals treated with a single dose of both compounds. Similar to *in vitro* findings, p-CDK1 Y15 levels were decreased in response to either monotherapy, and the decrease was more profound when the treatments were combined (Fig. 3D). In contrast to *in vitro* findings, some tumors displayed rapid reactivation of p-ERK1/2 and p-S6 in response to treatment, consistent with reported data (43). Although p-S6 seemed to be inhibited by azenosertib in the NCI-H2122 xenograft model, this effect was not observed in the *in vitro* analyses of NCI-H2122 (Fig. 1B), MIA PaCa-2, SW837 (Supplementary Fig. S2B), or NCI-H23 (Fig. 2C), suggesting that this may be a xenograft-specific phenotype. Likewise, we observed the same p-S6 decrease in azenosertib-treated SW1573 xenografts (Supplementary Fig. S3B) but not when the cell line was treated *in vitro* (Supplementary Fig. S3A), further corroborating that this is likely a phenomenon in xenografts. Critically, γH2AX and c-Casp-3/7 were only slightly upregulated in response to the monotherapies but highly increased in response to combination treatment (Fig. 3D). Further tumor analysis demonstrates significantly reduced proliferation by Ki67 IHC and increased cell death in response to combination treatment relative to either monotherapy (Supplementary Fig. S4).

These data demonstrate that although treatment with azenosertib or sotorasib is moderately efficacious on its own, combining both drugs enhances efficacy and can lead to tumor regression in the NCI-H2122 model.

### Combination of azenosertib with *KRAS*^G12C^ inhibitors improves efficacy and drives tumor regression in models of colorectal cancer and PDAC

**Figure 4:**
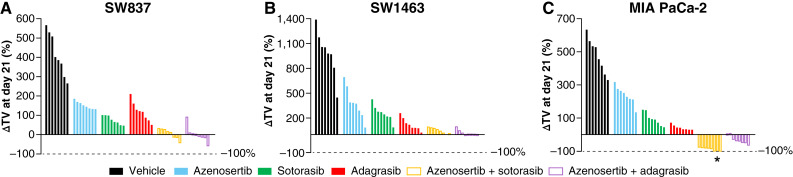
Combination of azenosertib with *KRAS*^G12C^ inhibitors improves efficacy and drives tumor regression in models of colorectal cancer and PDAC. **A,** Percent change in tumor volume (ΔTV) of individual SW837 xenografts in NOD/SCID mice on day 21 of treatment (*n* = 8/group). Azenosertib dosed at 60 mg/kg orally every day. Sotorasib and adagrasib dosed at 30 mg/kg orally every day. **B,** ΔTV of individual SW1463 xenografts in NCG mice on day 21 of treatment (*n* = 8/group). Azenosertib dosed at 60 mg/kg orally every day. Sotorasib and adagrasib dosed at 30 mg/kg orally every day. **C,** ΔTV of individual MIA PaCa-2 xenografts in BALB/c nude mice on day 21 of treatment (*n* = 8/group). Azenosertib dosed at 80 mg/kg orally every day. Sotorasib and adagrasib dosed at 10 mg/kg orally every day. *, complete regression (3/8). **A–C,** Values below 0 indicate regression. *P* < 0.0001 for all comparisons with vehicle; *P* < 0.0001 for all combinations compared with respective monotherapies except SW1463 adagrasib vs. combo (*P* = 0.67). NSG, NOD/SCID gamma.

To determine if *in vitro* efficacy across tumor types was consistent *in vivo*, we evaluated the combination activity in CDX models of colorectal cancer (SW837 and SW1463) or PDAC (MIA PaCa-2). As observed in NCI-H2122, monotherapy treatment resulted in moderate but statistically significant (*P* < 0.0001) TGI when compared with vehicle. Importantly, combination treatment with either sotorasib or adagrasib resulted in deeper average TGI (99% and 100% in SW837; 94% and 99% in SW1463; 118% and 107% in MIA PaCa-2, respectively; *P* < 0.0001) when compared with either monotherapy in all three models (Supplementary Fig. S5A–S5C). Furthermore, individual animals experienced tumor regression in combination arms in SW837 (sotorasib 3/8; adagrasib 5/8), SW1463 (sotorasib 1/8; adagrasib 4/8), and MIA PaCa-2 (sotorasib 8/8; adagrasib 7/8; Fig. 4A–C). Additionally, three of eight (38%) of the animals in the combination arm of the MIA PaCa-2 study experienced complete tumor regression (Fig. 4C). This combination was well tolerated across all tested models (Supplementary Fig. S6A–S6C).

These data demonstrate that the combination of azenosertib with *KRAS*^G12C^ inhibitors drives significantly increased TGI relative to monotherapy and high rates of regression in tumor models of colorectal cancer and PDAC. Only minor differences were observed between sotorasib and adagrasib, confirming that inhibition of KRAS and its downstream activity are the mechanistic basis for synergy with azenosertib.

### Combination of azenosertib with *KRAS*^G12C^ inhibitors *in vivo* increases the depth and duration of TGI in NSCLC models sensitive to *KRAS*^G12C^ inhibition

**Figure 5:**
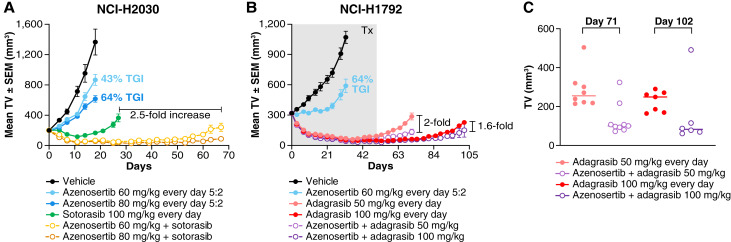
Combination of azenosertib with *KRAS*^G12C^ inhibitors *in vivo* increases the depth and duration of TGI in NSCLC models sensitive to *KRAS*^G12C^ inhibition. **A,** Mean tumor volume ± SEM of subcutaneous NCI-H2030 xenografts in NOD/SCID mice (*n* = 9/group). Azenosertib and sotorasib monotherapy arms ended on days 18 and 27, respectively. Combination groups remained on-treatment until day 67. *P* < 0.0001 for all comparisons with vehicle; *P* < 0.0001 for combinations compared with azenosertib monotherapy at day 18; *P* < 0.0001 for combinations compared with sotorasib monotherapy at day 26. **B,** Mean tumor volume ± SEM of subcutaneous NCI-H1792 xenografts in NOD/SCID mice (*n* = 9/group). Azenosertib monotherapy and adagrasib 50 mg/kg monotherapy and combination arms were treated for 32 days. Adagrasib 100 mg/kg monotherapy and combination arms continued dosing until day 53, and then animals were monitored off-treatment until day 71 or 102. *P* < 0.0001 for comparison of azenosertib with vehicle; *P* < 0.0001 for combinations and adagrasib monotherapy compared with azenosertib monotherapy at day 32. **C,** Individual animal tumor volumes on day 71 or 102 for adagrasib monotherapy (red) and combination (purple) from B. Two animals in the 100 mg/kg adagrasib monotherapy group and three animals in the 100 mg/kg adagrasib combination group were removed before day 102 because of thymoma development unrelated to treatment.

Although TGI and regression are important aspects of antitumor activity, durable responses to therapy are equally important and remain a critical unmet need in patients with *KRAS*^G12C^-mutant cancers. To assess whether the addition of azenosertib extends the duration of response to *KRAS*^G12C^ inhibitors, we evaluated the combination in the NCI-H2030 and NCI-H1792 NSCLC CDX models. In response to azenosertib monotherapy, the NCI-H2030 CDX model exhibited moderate, dose-dependent TGI that was statistically significant relative to vehicle (*P* < 0.0001). Sotorasib monotherapy displayed early regression but rapid recurrence and progression within 27 days. Intriguingly, combining sotorasib with azenosertib not only increased the depth of response but extended the time until progression by at least 2.5-fold (Fig. 5A) and further resulted in a significant increase in median survival (*P* = 0.007, Supplementary Fig. S7A). Additionally, the combination of azenosertib and sotorasib continued to be well tolerated throughout the extended treatment period of 67 days (Supplementary Fig. S6D).

In the NCI-H1792 NSCLC model, azenosertib monotherapy resulted in moderate but statistically significant TGI relative to vehicle (*P* < 0.0001). Although adagrasib alone resulted in durable regression, tumors rapidly regrew in the low-dose adagrasib (50 mg/kg every day) group when treatment was withdrawn (Fig. 5B). In contrast, tumor regression was maintained in seven of nine (78%) animals in the corresponding combination group (Fig. 5C). Additionally, high-dose adagrasib (100 mg/kg every day) extended the duration of regression longer than the lower dose, yet tumor regrowth was eventually observed in all animals by the end of the study (Fig. 5B). Combination treatment at the higher dose again maintained near-complete regression in five of six (83%) animals at the end of the study (Fig. 5C). Furthermore, both combination arms significantly extended the median survival over monotherapy treatment at either adagrasib dose (Supplementary Fig. S7B). The combination was well tolerated at all doses during the treatment period as illustrated by minimal body weight loss (Supplementary Fig. S6E).

These data establish that the combination of azenosertib with *KRAS*^G12C^ inhibition can extend the depth and duration of response in NSCLC models which are already relatively sensitive to *KRAS*^G12C^ inhibitors, effectively delaying the onset of resistance.

### Combination of azenosertib with *KRAS*^G12C^ inhibitors shows efficacy in models of intrinsic and acquired resistance to G12C inhibitors *in vivo*

**Figure 6:**
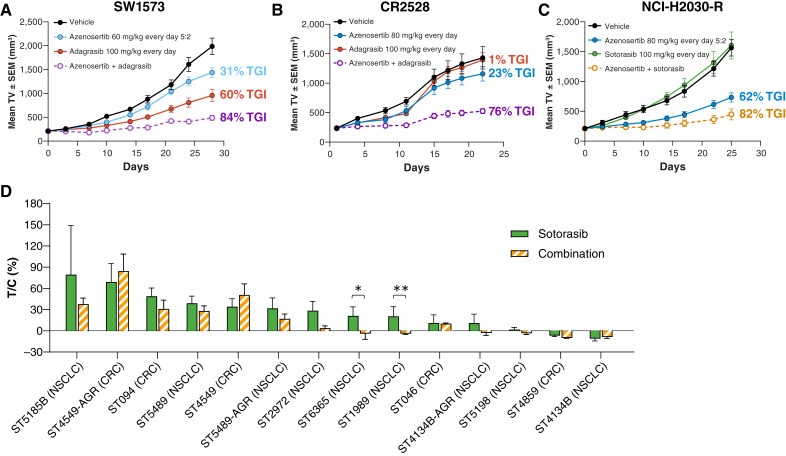
Combination of azenosertib with *KRAS*^G12C^ inhibitors shows efficacy in models of innate and acquired resistance to G12C inhibitors *in vivo*. **A,** Mean tumor volume ± SEM of subcutaneous SW1573 xenografts in NOD/SCID mice (*n* = 9/group). **B,** Mean tumor volume ± SEM of subcutaneous CR2528 PDX in BALB/c nude mice treated for 24 days (*n* = 8/group). **C,** Mean tumor volume ± SEM of subcutaneous NCI-H2030-R xenografts in NOD/SCID mice (*n* = 9/group). Xenografts were passaged and grown in mice maintained on 100 mg/kg sotorasib until grouping. **A–C, ***P* < 0.0001 for all comparisons with vehicle; *P* < 0.0001 for combinations compared with monotherapies. **D,** Percent T/C of subcutaneous PDX models implanted into athymic nude [Crl:NU(NCr)-Foxn1^nu^] mice (*n* = 3/group). Animals were treated for up to 28 days with the indicated compounds. *, *P* < 0.05; **, *P* < 0.001. CRC, colorectal cancer; T/C, treated/control; TV, tumor volume.

Many patients respond to *KRAS*^G12C^ inhibition; however, a substantial proportion of patients are inherently refractory (9, 10). We thus sought to demonstrate combination activity in models that are inherently resistant (≤60% TGI; ref. 44) to *KRAS*^G12C^ inhibition. The SW1573 model displayed resistance to sotorasib and adagrasib *in vitro* (Fig. 1A) and resulted in only 60% TGI in response to 100 mg/kg adagrasib (Fig. 6A). This model is also resistant to azenosertib, as the monotherapy dose of 60 mg/kg every day 5:2 resulted in a relatively weak response of 31% TGI. Interestingly, combination treatment was significantly more efficacious than either monotherapy (*P* < 0.0001) resulting in 84% TGI and was well tolerated (Supplementary Fig. S6F).

To test this combination in a more clinically relevant model of intrinsic resistance, we utilized the CR2528 PDX model of colorectal cancer. CR2528 exhibited resistance to both azenosertib and adagrasib monotherapy at high doses, with a TGI of 23% and 1%, respectively. Even in this highly resistant model, the combination led to approximately threefold increased TGI (76%, *P* < 0.0001) compared with azenosertib monotherapy (Fig. 6B) and was well tolerated (Supplementary Fig. S6G). These data demonstrate that tumor models bearing some level of intrinsic resistance to azenosertib monotherapy or *KRAS*^G12C^ inhibitors may benefit from the combination.

Although some patients are intrinsically refractory to *KRAS*^G12C^ inhibition, many will acquire resistance in response to treatment. To determine if the combination can drive TGI in a model with acquired resistance to *KRAS*^G12C^ inhibition, we tested sotorasib-resistant NCI-H2030 xenografts that were derived through continuous exposure to sotorasib *in vivo* (hereafter referred to as NCI-H2030-R). As expected, the treatment of the NCI-H2030-R model was tolerated (Supplementary Fig. S5H) and displayed high levels of resistance to sotorasib monotherapy (Fig. 6C) while maintaining sensitivity to azenosertib similar to that of the parental line (Fig. 5A). Combination treatment resulted in a 20% increase in TGI (82% vs. 62%) with one animal experiencing tumor regression, although statistical significance was not achieved (*P* = 0.07).

To further assess how widely applicable this combination is in clinically translatable models, we conducted a mouse PDX screen in 14 NSCLC or colorectal cancer PDX models with varying responses to *KRAS*^G12C^ inhibition, including paired sensitive and resistant models derived via continuous exposure *in vivo*. Ten of 14 (71%) models derived additional benefit from combination therapy over monotherapy sotorasib, including statistically significant differences in the ST6365 and ST1989 models (*P* < 0.05; Fig. 6D). Furthermore, we observed combination benefit in two paired resistant models irrespective of relative sensitivity to sotorasib monotherapy.

These data show that the combination of azenosertib and *KRAS*^G12C^ inhibition is generally superior to monotherapy across a range of models with intrinsic and acquired resistance to *KRAS*^G12C^ inhibitors, both of which represent unmet clinical needs.

## Discussion

In this study, we evaluated the efficacy and synergistic effect of combining azenosertib with two FDA-approved *KRAS*^G12C^ inhibitors across a panel of cell lines and xenograft models representing tumor indications in which *KRAS*^G12C^ alterations are common. We demonstrated *in vitro* in 2D anchorage-dependent (Fig. 1) and 3D anchorage-independent (Fig. 2) models and in *in vivo* xenograft models (Figs. 3–6) of NSCLC, colorectal cancer, and PDAC that azenosertib shows both meaningful monotherapy potency and significant synergy with both sotorasib and adagrasib. Analysis of key PD biomarkers of DNA damage, RS (γH2AX), and apoptosis (c-Casp-3/7) further validated previously reported increases in RS in *KRAS*^G12C^ inhibitor–treated cancer cells while also demonstrating further upregulation in response to combination treatment. These PD changes suggest that dual inhibition of *KRAS*^G12C^ and WEE1 are driving DNA damage and RS through independent but complementary mechanisms to further increase cancer cell death via apoptosis.

Patients with *KRAS*^G12C^ alterations are known to have significantly worse prognosis than patients whose tumors are wild-type (WT) for KRAS (45, 46). The advent of novel *KRAS*^G12C^ inhibitors improved patient response rates and provided substantial clinical benefit in this historically hard-to-treat patient population. However, although most patients in these trials derived meaningful clinical benefit, the depth and duration of response could be enhanced to further improve clinical benefit. Our studies in CDX models of NSCLC, colorectal cancer, and PDAC that are already relatively sensitive to *KRAS*^G12C^ inhibition indicate that azenosertib not only has statistically significant activity on its own but combining it with *KRAS*^G12C^ inhibitors greatly improves antitumor efficacy, often resulting in meaningful tumor regression (Figs. 3 and 4). A key clinical challenge of *KRAS*^G12C^ inhibitor monotherapy is the relatively short progression-free survival (PFS). The CodeBreaK 200 phase III study in previously treated *KRAS*^G12C^-mutant NSCLC reported a moderate but statistically significant (*P* = 0.0017) increase in PFS in the sotorasib (5.6 months) versus docetaxel (4.5 months) arm and an overall survival that was not significantly different (10.6 vs. 11.3 months; *P* = 0.53; ref. 47). Although this represents clinically meaningful progress in treatment opportunities for patients with *KRAS*^G12C^ NSCLC, there remains a need to further extend this duration of response. Our *in vivo* data (Fig. 5) suggests that combining azenosertib with *KRAS*^G12C^ inhibition may enhance the efficacy of *KRAS*^G12C^ inhibitors and ultimately extend PFS in patients with *KRAS*^G12C^ NSCLC.

Although monotherapy with *KRAS*^G12C^ inhibitors have demonstrated clear clinical benefit in patients with *KRAS*^G12C^ mutations, a substantial portion of these patients do not respond (9, 10). Additionally, there is a disparity in the clinical responses across tumor types. Patients with *KRAS*^G12C^ NSCLC exhibit robust response rates to *KRAS*^G12C^ monotherapy; however, patients with *KRAS*^G12C^ colorectal cancer or PDAC respond at much lower frequencies (9, 48, 49). To increase the response rates across indications, many combination treatment strategies have been devised; however, the majority of these strategies rely on vertical pathway inhibition to further block signaling both upstream and downstream of KRAS (13). Targeting feedback activation of receptor tyrosine kinase has demonstrated benefit in colorectal cancer (50). However, clinical studies evaluating the effects of targeting multiple downstream components (e.g., MEK and ERK) have failed largely because of overlapping on-target toxicity (51, 52), highlighting a need for alternative strategies which could provide similar or better clinical results with less risk for overlapping toxicities. Here, we show synergy between azenosertib and *KRAS*^G12C^ inhibitors *in vivo* in *KRAS*^G12C^ CDX and PDX models of colorectal cancer and PDAC, highlighting the activity across indications which have variable responses to *KRAS*^G12C^ inhibition in the clinic (Figs. 4 and 6). Some of these models are intrinsically resistant to both inhibitors as monotherapies, and yet we find that TGI can be further increased with the combination (Fig. 6). The most commonly reported adverse events in response to *KRAS*^G12C^ inhibitors include diarrhea, vomiting, and elevated liver enzymes (9). In contrast, serious adverse events observed in response to WEE1 inhibitors, which we and others have reported, are largely hematologic (e.g., anemia, neutropenia, and thrombocytopenia; ref. 53, 54). Based on these observed clinical safety signals, the risk of overlapping toxicity may be reduced, supported by the observation that this combination is well tolerated across all tested CDX and PDX tumor models (Fig. 3; Supplementary Fig. S6).

In addition to patients that are inherently less responsive to *KRAS*^G12C^ monotherapy, even those that initially respond to treatment eventually relapse. There is a diversity of reported resistance mechanisms across both preclinical and clinical datasets, but many highlight mutations in other members of the MAPK and PI3K signaling cascades or upstream activation of receptor tyrosine kinase signaling (55, 56). These mutations either reactivate signaling through KRAS or bypass KRAS by activating parallel pathways that drive cancer cell growth but in either case may increase RS and DNA damage. Thus, these resistant tumors may be sensitive to azenosertib monotherapy and vulnerable to combination treatment with azenosertib and *KRAS*^G12C^ inhibitors. Our data show that acquired resistance models can be sensitive to azenosertib and display synergistic TGI with combination treatment (Fig. 6). We have shown this effect to be generally consistent across both CDX and PDX models of NSCLC and colorectal cancer, though unsurprisingly responses observed in the larger PDX screen proved to be less clear (Fig. 6D). Both the inherent heterogeneity of PDX models and the small test size (*n* = 3 mice/group) likely contributed to the variability. Taken together, these data provide a rationale for future clinical studies of synergistic combinations, including with azenosertib, to include patients that have been pretreated with *KRAS*^G12C^ inhibitors.

Interestingly, detailed biomarker analyses of patients receiving *KRAS*^G12C^ inhibitor monotherapy have shown that genetic mutations beyond *KRAS*^G12C^ may play a role in response to *KRAS*^G12C^ inhibition. Specifically in NSCLC, mutations in genes including *KEAP1*, *SMARCA4*, and *CDKN2A* have been associated with resistance to *KRAS*^G12C^ inhibitors (9, 10, 57), and the mutation in *STK11* has been associated with sensitivity to *KRAS*^G12C^ inhibition (9, 10). Furthermore, a prior study with the WEE1 inhibitor adavosertib (AstraZeneca) identified preclinical efficacy in STK11-deficient NSCLC (58), providing additional rationale for combining azenosertib and *KRAS*^G12C^ inhibition in tumors with this genotype. A recently published study identified WEE1 as a synthetic lethal target in *KRAS*^G12C^-mutated NSCLC through a targeted CRISPR-Cas9 screen (32). This orthogonal approach validates the key role of WEE1 in maintaining cell viability in this cancer type. The authors identified *TP53* mutation as a key co-mediator of sensitivity to WEE1 inhibition; however, this effect was not shown to be *KRAS*^G12C^-specific. The association with *TP53* mutation and WEE1 inhibitor sensitivity was also identified in a separate clinical study of *KRAS*-mutant colorectal cancer (31). In our study, we observed a trend that the majority of models experiencing tumor regression greater than *KRAS*^G12C^ inhibitor monotherapy in response to the combination had co-mutation of *TP53* and *KRAS*^G12C^ (NCI-H2030, NCI-H1792, NCI-H2122, SW837, SW1463, MIA PaCa-2, ST4859, and ST6365), whereas the inherently resistant models (SW1573 and CR2528) were *TP53* WT (Supplementary Table S2). However, these two *TP53* WT models also harbored *PIK3CA* mutations, which may activate downstream PI3K signaling even in the presence of *KRAS*^G12C^ inhibitors and could explain the relative resistance to *KRAS*^G12C^ monotherapy and combination treatment. Still, some models without *TP53* mutation (ST5198 and ST1989) also experienced regression greater than *KRAS*^G12C^ inhibitor monotherapy in response to the combination. Given the confounding responses in the presence or absence of *TP53* mutation and the relatively low number of models tested, the present data are insufficient to make statistically significant associations, and further investigation of the role of various co-mutations on WEE1/*KRAS*^G12C^ inhibitor combination therapy is warranted.

*KRAS* ^G12C^ mutation is frequently observed across many tumor types, yet *KRAS*^G12C^ is only a fraction of the overall *KRAS*-mutant patient population (59). Indeed, mutations are variable and include other codon 12 mutations such as G12D/V/A, as well as other activating mutations in codons 13 and 61, all of which drive activation of downstream KRAS signaling. The KRAS inhibitor landscape has expanded in recent years to include inhibitors specific to other non-G12C mutations as well as pan-RAS inhibitors that target WT and mutant KRAS, as well as NRAS and HRAS (41, 59). In contrast to sotorasib and adagrasib which are RAS(off) inhibitors, RAS(on) inhibitors can block downstream signaling through blocking KRAS in the GTP-bound on state. All these inhibitors have the same net effect, which is to block downstream signaling through MAPK/PI3K and inhibit tumor cell growth. Given this shared phenotype, the results which we present here demonstrate a rationale which may be applied to the combination of azenosertib with any available RAS inhibitor, regardless of the binding mechanism or RAS isoform specificity. Future preclinical combination studies will more extensively test the combination of azenosertib with these inhibitors, and we would anticipate additional tumor cell growth inhibition in combination with any of these alternative RAS inhibitors in RAS-driven tumors.

Although we have extensively validated WEE1 inhibition as synergistic with *KRAS*^G12C^ inhibition, inhibitors of different cell-cycle proteins (e.g., aurora kinase, PLK, CDK4/6, etc.) are currently in development and may also synergize with *KRAS*^G12C^ inhibition. Combinations of aurora kinase inhibitors with various inhibitors of the MAPK pathway, including EGFR (60), MEK (61), and *KRAS*^G12C^ (62), have demonstrated preclinical efficacy, resulting in initiation of a phase I study of an aurora kinase A inhibitor combined with sotorasib (NCT05374538). Extended G_1_ arrest has been reported to increase RS (63), and upregulation of PI3K/MAPK signaling is involved in resistance mechanisms to CDK4/6 inhibition (64, 65). These observations suggest that CDK4/6 inhibition could result in synergy with *KRAS*^G12C^ inhibition as CDK4/6 inhibitors are reported to arrest cells in G_1_. Based on supporting preclinical data (66), the combination of CDK4/6 inhibition with palbociclib and ERK1/2 inhibition with ulixertinib was tested in a phase Ib clinical trial (NCT03454035). Although the combination was determined to be safe, of the 26 patients treated only two patients demonstrated stable disease, and no responses were observed (67). These data suggest that although ERK inhibition is unlikely to provide significant clinical benefit in combination with CDK4/6 inhibition, additional studies should be done to more comprehensively evaluate combination with *KRAS*^G12C^ inhibitors. PLK1 is directly involved in phosphorylation and activation of WEE1 (68), and inhibition of PLK1 has been demonstrated to induce cell-cycle and mitotic defects (69). Moreover, preclinical and clinical data suggest that PLK1 inhibition is effective in *KRAS*-mutant colorectal cancer (32, 70), providing a rationale for more investigation on the combination efficacy with *KRAS*^G12C^ inhibition. Further preclinical studies are needed to evaluate the combination of *KRAS*^G12C^ inhibition with other cell-cycle inhibitors to determine if cell-cycle dysregulation is a general vulnerability in KRAS-driven cancers.

Overall, our results demonstrate significant combination activity of azenosertib and the *KRAS*^G12C^ inhibitors sotorasib and adagrasib in clinically relevant models of NSCLC, colorectal cancer, and PDAC and support continued evaluation of this combination in future preclinical and clinical studies.

## Supplementary Material

Supplementary Figure 1 Combination of azenosertib and KRASG12C inhibitors demonstrates synergy in NSCLC in 2D in vitro cellular assays by Bliss Independence model.

Supplementary Figure 2 Combination of azenosertib and KRASG12C inhibitors or WEE1 knockdown demonstrates synergy in CRC, PDAC, and NSCLC in 2D cellular assays

Supplementary Figure 3 Treatment of an NSCLC model with azenosertib + adagrasib results in biomarker changes in vitro and in vivo.

Supplementary Figure 4 Treatment of an NSCLC model with azenosertib + sotorasib results in reduced proliferation and minor histological changes in vivo.

Supplementary Figure 5 Combination of azenosertib with KRASG12C inhibitors improves efficacy and drives tumor regression in models of CRC and PDAC

Supplementary Figure 6 Treatment of CDX and PDX models with azenosertib + KRASG12C inhibitors is well tolerated in vivo

Supplementary Figure 7 Combination of azenosertib With KRASG12C inhibitors in vivo increases median survival in NSCLC models sensitive to KRASG12C inhibition

Supplementary Table 1 Antibodies used for this study.

Supplementary Table 2 TP53 status and pathogenicity for each model utilized for this study. Mutation status was derived from DepMap (https://depmap.org/portal/) for cell lines or provided by the PDX CRO.

Supplementary Data Figure Legends Supplementary Data Figure Legends
