# Supplementary figures and images for: The Selective WEE1 Inhibitor Azenosertib Shows Synergistic Antitumor Activity with KRASG12C Inhibitors in Preclinical Models
Source: Cancer Res Commun. 2025 Feb 5;5(2):240–52. doi: 10.1158/2767-9764.CRC-24-0411 (PMC11795354; doi:10.1158/2767-9764.CRC-24-0411)

Supplementary Figure 1

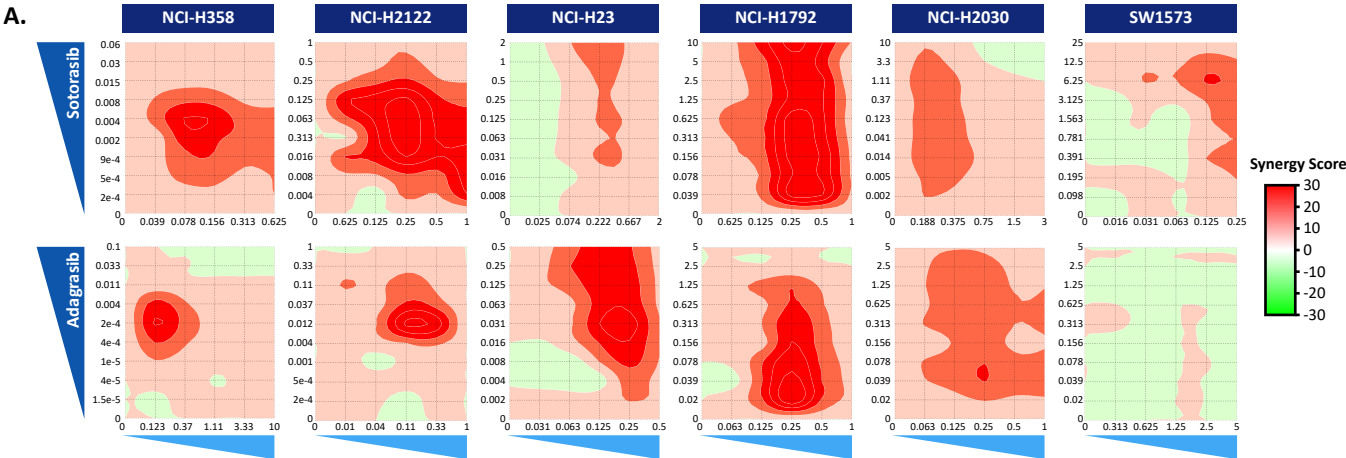

Supplement: Supplementary Figure 1 — Combination of azenosertib and KRASG12C inhibitors demonstrates synergy in NSCLC in 2D in vitro cellular assays by Bliss Independence model. [file crc-24-0411_supplementary_figure_1_suppsf1.pdf]

Supplementary Figure 2

A.

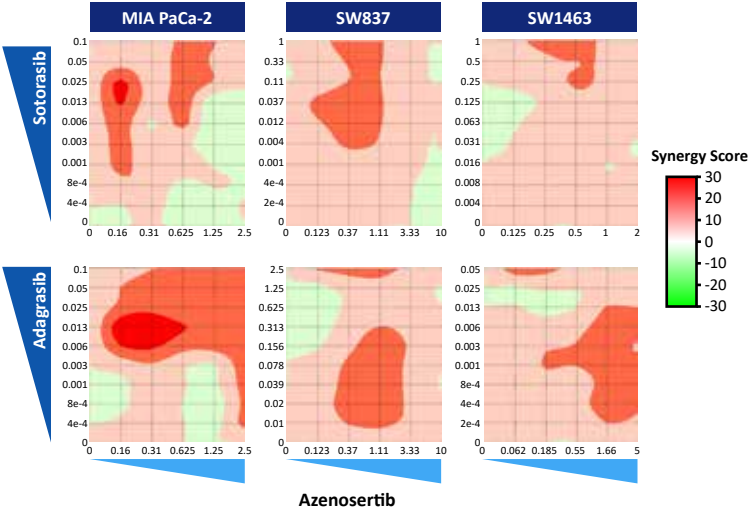

B.

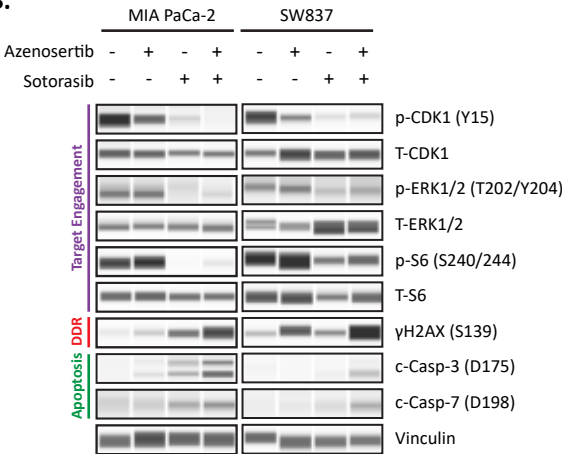

C.

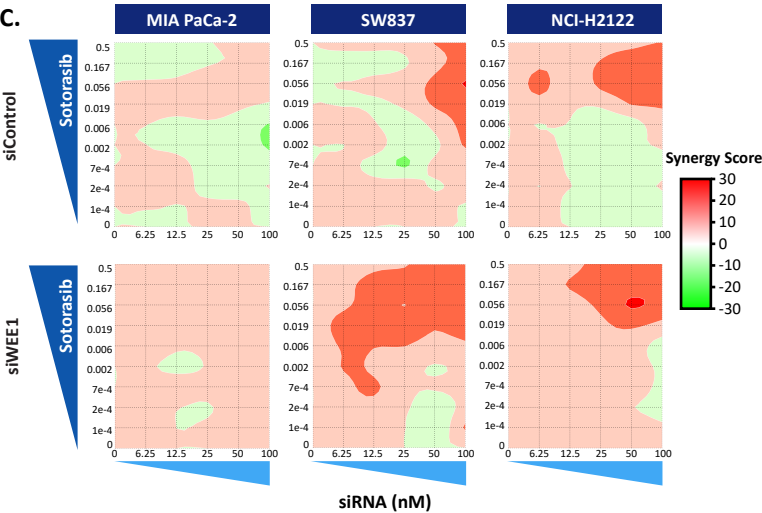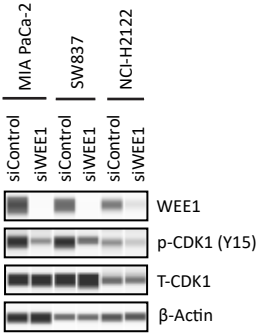

D.

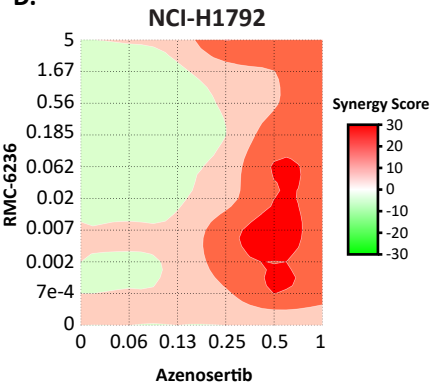

Supplement: Supplementary Figure 2 — Combination of azenosertib and KRASG12C inhibitors or WEE1 knockdown demonstrates synergy in CRC, PDAC, and NSCLC in 2D cellular assays [file crc-24-0411_supplementary_figure_2_suppsf2.pdf]

Supplementary Figure 3

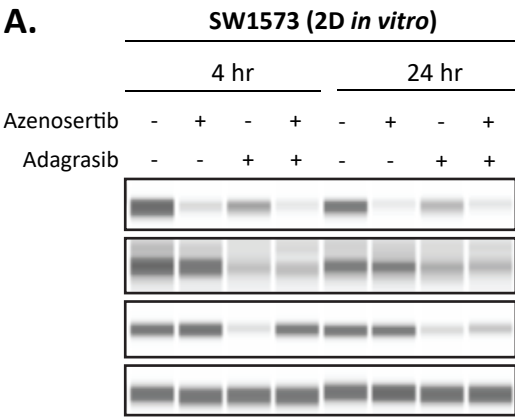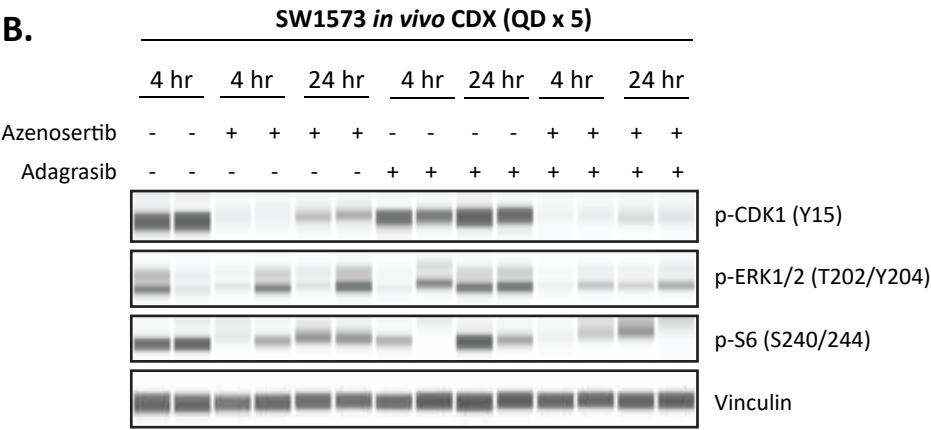

Supplement: Supplementary Figure 3 — Treatment of an NSCLC model with azenosertib + adagrasib results in biomarker changes in vitro and in vivo. [file crc-24-0411_supplementary_figure_3_suppsf3.pdf]

**Supplementary Figure 4**

**A.**

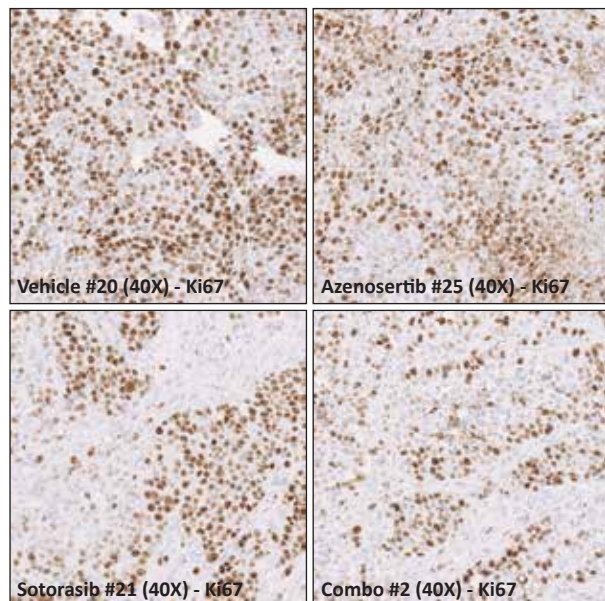

**B.**

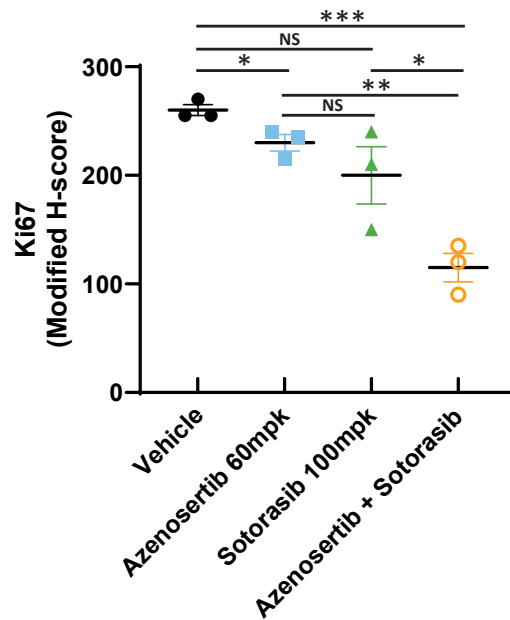

**C.**

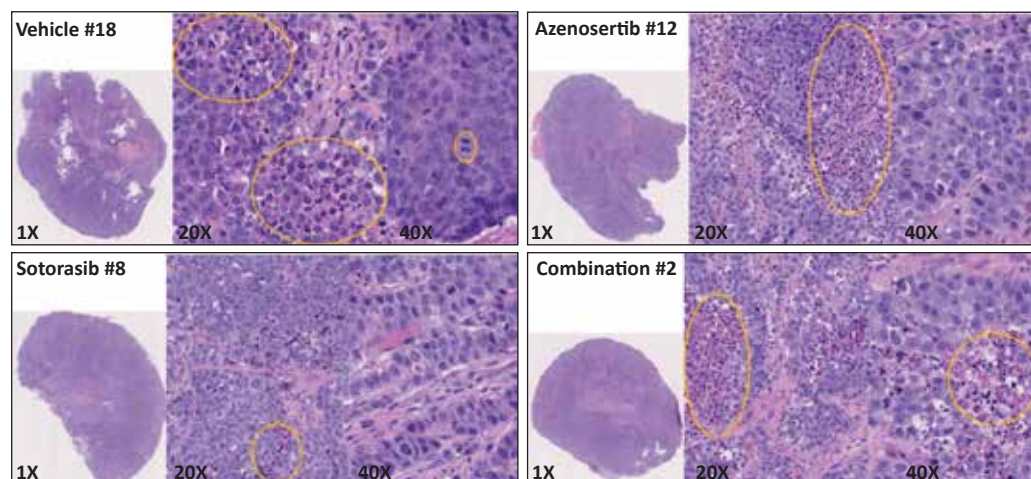

**D.**

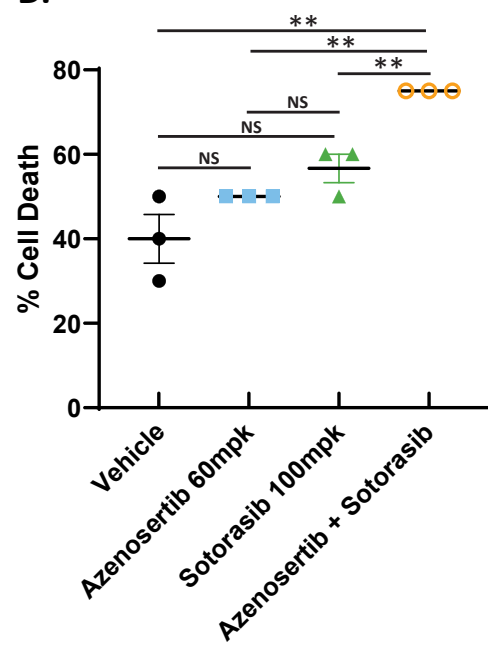

Supplement: Supplementary Figure 4 — Treatment of an NSCLC model with azenosertib + sotorasib results in reduced proliferation and minor histological changes in vivo. [file crc-24-0411_supplementary_figure_4_suppsf4.pdf]

Supplementary Figure 5

A.

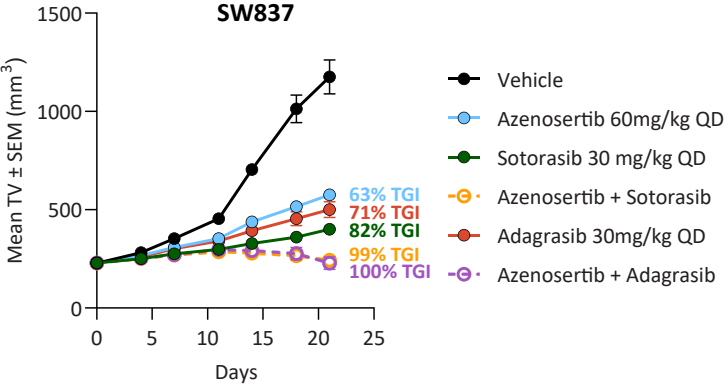

B.

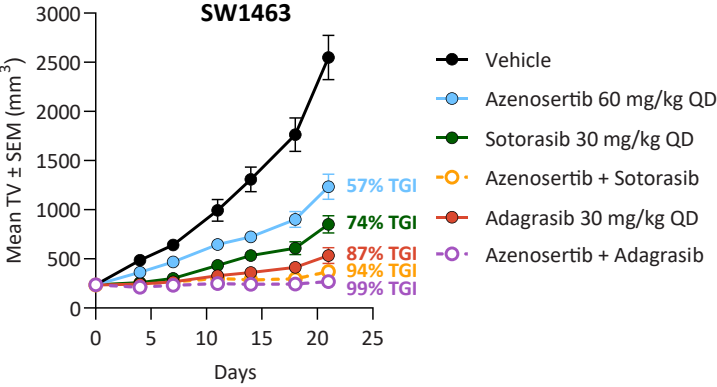

C.

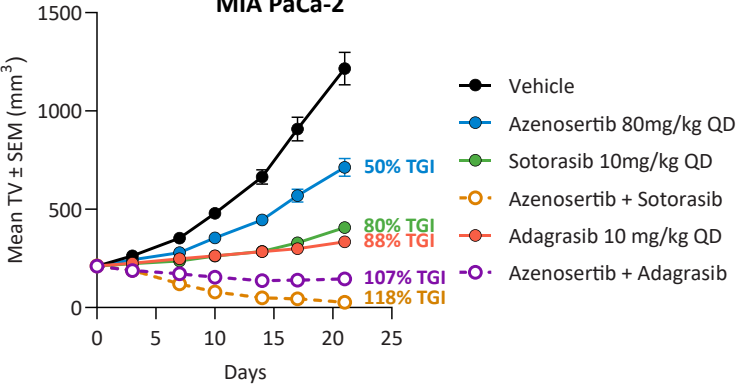

Supplement: Supplementary Figure 5 — Combination of azenosertib with KRASG12C inhibitors improves efficacy and drives tumor regression in models of CRC and PDAC [file crc-24-0411_supplementary_figure_5_suppsf5.pdf]

Supplementary Figure 6

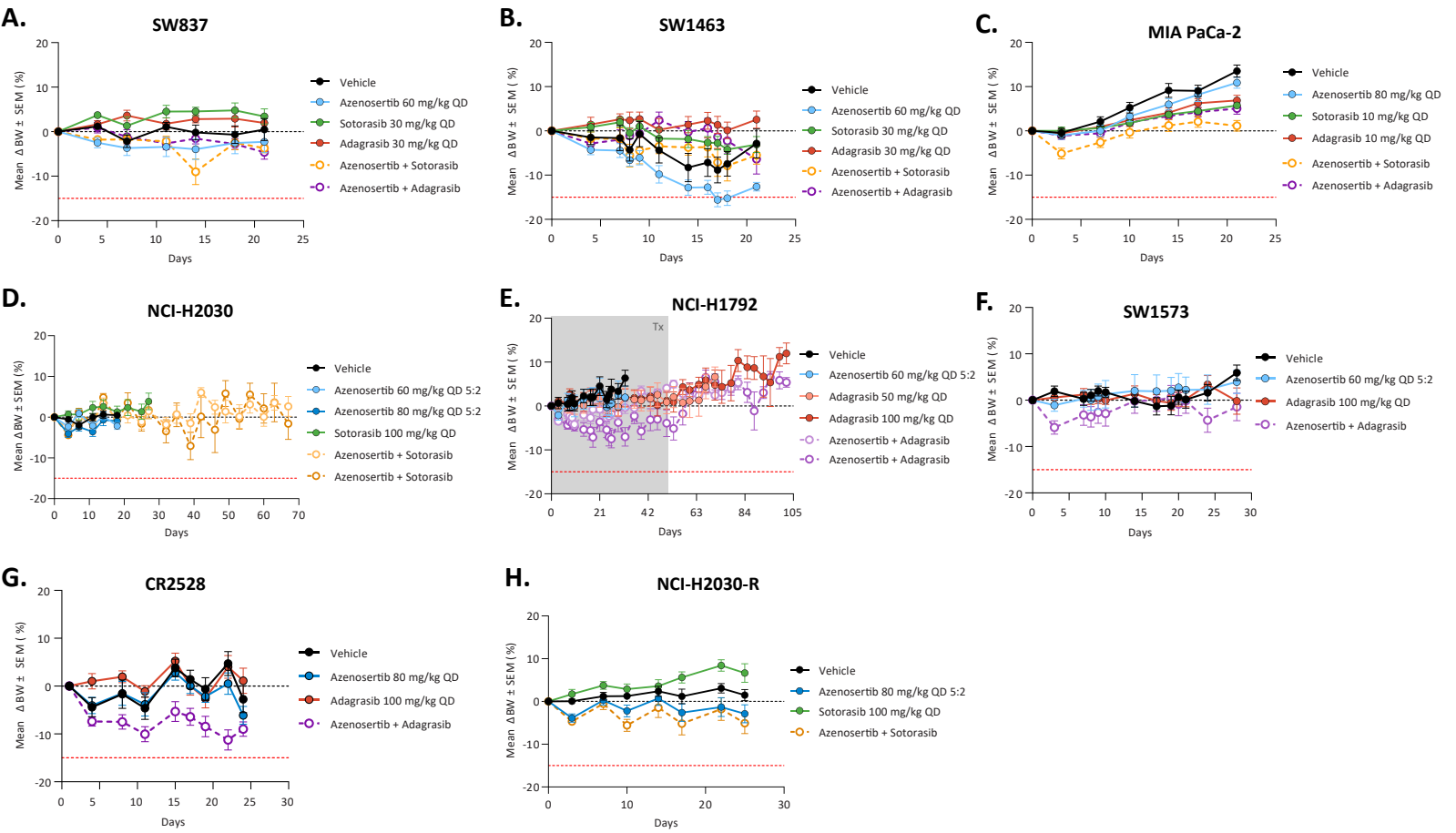

Supplement: Supplementary Figure 6 — Treatment of CDX and PDX models with azenosertib + KRASG12C inhibitors is well tolerated in vivo [file crc-24-0411_supplementary_figure_6_suppsf6.pdf]

Supplementary Figure 7

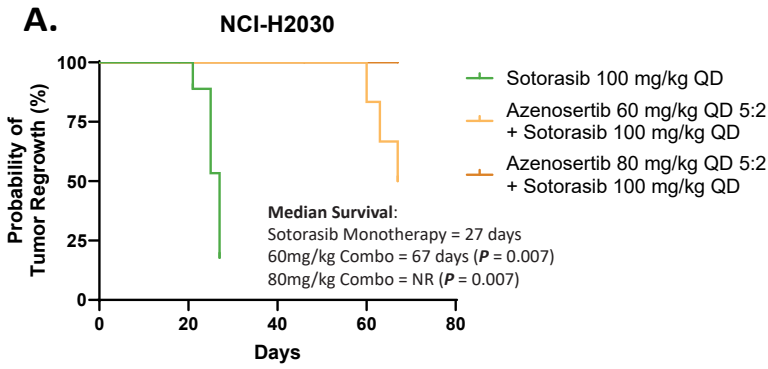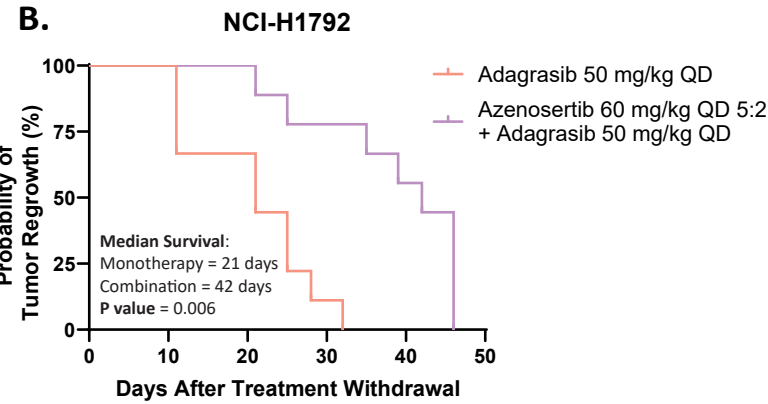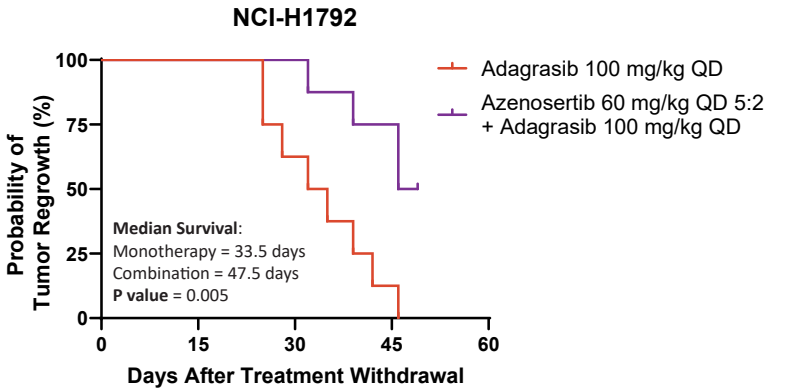

Supplement: Supplementary Figure 7 — Combination of azenosertib With KRASG12C inhibitors in vivo increases median survival in NSCLC models sensitive to KRASG12C inhibition [file crc-24-0411_supplementary_figure_7_suppsf7.pdf]
