# Supplementary material for: The Selective WEE1 Inhibitor Azenosertib Shows Synergistic Antitumor Activity with KRASG12C Inhibitors in Preclinical Models
Source: Cancer Res Commun. 2025 Feb 5;5(2):240–52. doi: 10.1158/2767-9764.CRC-24-0411 (PMC11795354; doi:10.1158/2767-9764.CRC-24-0411)
Supplement: Supplementary Data Figure Legends [file crc-24-0411_supplementary_data_figure_legends_suppsfl1-sfl7.docx]

**SUPPLEMENTARY FIGURE LEGENDS**

**Supplementary Figure 1.** Combination of azenosertib and KRAS^G12C^ inhibitors demonstrates synergy in NSCLC in 2D *in vitro* cellular assays by Bliss Independence model. **A.** 7-day combination treatment dose matrices in KRAS^G12C^ NSCLC cell lines cultured in 2D. Bliss independence model is depicted where scores ≥ 10 are synergistic. Doses are in µM. Cell lines are arranged from most sensitive to least sensitive to KRAS^G12C^ monotherapy (left to right).

**Supplementary Figure 2.** Combination of azenosertib and KRAS^G12C^ inhibitors demonstrates synergy in CRC and PDAC and when WEE1 is targeted genetically in 2D cellular assays. **A.** 7-day combination treatment dose matrices in KRAS^G12C^ CRC and PDAC cell lines cultured in 2D. Loewe additivity model is depicted where scores ≥ 10 are synergistic. Doses are in µM. Cell lines are arranged from most sensitive to least sensitive to KRAS^G12C^ monotherapy (left to right). **B.** Western blot of protein expression from MIA PaCa-2 PDAC cells treated with DMSO, 310nM azenosertib, 12.5nM sotorasib, or the combination, and SW837 CRC cells treated with DMSO, 200nM azenosertib, 10nM sotorasib, or the combination for 24 hours. **C.** (Left) 7-day combination treatment dose matrices in KRAS^G12C^ cell lines cultured in 2D. Loewe additivity model is depicted where scores ≥ 10 are synergistic. Pooled siRNA is a mixture of 4 siRNA’s. (Right) Western blot of protein expression from indicated cell lines treated with 100nM siControl or siWEE1 for 48 hours. **D.** 7-day combination treatment dose matrix in the NCI-H1792 cell line cultured in 2D. Doses are in µM.

**Supplementary Figure 3.** Treatment of an NSCLC model with azenosertib + adagrasib results in biomarker changes *in vitro* and *in vivo*. **A.** Western blot of protein expression from SW1573 cells treated with DMSO, 625nM azenosertib, 1.25µM adagrasib, or a combination of both for 4h or 24h. **B.** Western blot of protein expression from SW1573 tumors treated with five daily doses of the indicated compounds.

**Supplementary Figure 4.** Treatment of an NSCLC model with azenosertib + sotorasib results in reduced proliferation and minor histological changes *in vivo*. **A.** Representative images of Ki67 IHC performed on NCI-H2122 tumors treated with one dose of the indicated compounds. **B.** Modified Ki67 H-scores of NCI-H2122 tumors treated with one dose of the indicated compounds. * = p < 0.05; ** = p < 0.001; *** p < 0.0001. **C.** Representative images of H&E staining performed on NCI-H2122 tumors treated with one dose of the indicated compounds. [Upper Left] Pink areas in the 1X image are large areas of cell death; smaller areas of cell death are in the 20X panel (circled). The 40X panel has a sheet of tumor cells with a prominent mitotic figure (small circle). [Upper right] The 20X panel has an area of cell death (circle). [Lower Left] The 20X panel has numerous individual dead cells and small clusters (circle). The 40X panel shows the more epithelial/gland-like tumor cell arrangement. [Lower Right] The 20X and 40X panels have larger foci of dead tumor cells (circles). D. Percent cell death of NCI-H2122 tumors treated with one dose of the indicated compounds as scored by a board-certified veterinary pathologist with experience in laboratory animals and toxicologic pathology. ** = p < 0.001.

**Supplementary Figure 5.** Combination of azenosertib with KRAS^G12C^ inhibitors improves efficacy and drives tumor regression in models of CRC and PDAC**. A.** Mean tumor volume ± SEM of subcutaneous SW837 xenografts in NOD/SCID mice treated for 21 days (n=8/group). **B.** Mean tumor volume ± SEM of subcutaneous SW1463 xenografts in NCG mice treated for 21 days (n=8/group). **C.** Mean tumor volume ± SEM of subcutaneous MIA PaCa-2 xenografts in BALB/c nude mice treated for 21 days (n=8/group). **A-C.** p<0.0001 for all comparisons to vehicle, p<0.0001 for all combinations compared to respective monotherapies except SW1463 adagrasib vs combo (p = 0.67).

**Supplementary Figure 6.** Treatment of CDX and PDX models with azenosertib + KRAS^G12C^ inhibitors is well tolerated *in vivo*. **A-H.** Mean percent change in body weight from day 0 (ΔBW). **A.** SW837 xenografts in NOD/SCID mice treated for 21 days (n=8/group). **B.** SW1463 xenografts in NCG mice treated for 21 days. **C.** MIA PaCa-2 xenografts in BALB/c nude mice treated for 21 days. **D.** NCI-H2030 xenografts in NOD/SCID mice (n=9/group). Azenosertib and sotorasib monotherapy arms ended day 18 and 27, respectively. Combination groups remained on treatment until day 67. **E.** NCI-H1792 xenografts in NOD/SCID mice (n=9/group). Azenosertib monotherapy was treated for 32 days. Adagrasib and combination arms continued dosing until day 53, then animals were monitored off‑treatment until day 71 or day 102. **F.** SW1537 xenografts in NOD/SCID mice treated for 28 days (n = 9/group). **G.** CR2528 patient-derived xenografts in BALB/c nude mice treated for 24 days (n=8/group). **H.** NCI-H2030-R xenografts in NOD/SCID mice treated for 25 days (n=9/group). Xenografts were passaged and grown in mice maintained on 100 mg/kg sotorasib until grouping.

**Supplemental Figure 7.** Combination of azenosertib With KRAS^G12C^ inhibitors *in vivo* increases median survival in NSCLC models sensitive to KRAS^G12C^ inhibition. **A.** Kaplan-Meier survival curve shows the probability of tumor regrowth defined as the time until tumor volume reaches ≥ 250mm^3^. NR = Not Reached. **B.** Kaplan-Meier survival curves show the probability of tumor regrowth defined as the time until tumor volume reaches ≥ 100mm^3^.

**Supplementary Table 1**. Antibodies used for this study.

**Supplementary Table 2**. *TP53* status and pathogenicity for each model utilized for this study. Mutation status was derived from DepMap (<https://depmap.org/portal/>) for cell lines or provided by the PDX CRO.
